# Stem cells-derived exosomes alleviate neurodegeneration and Alzheimer’s pathogenesis by ameliorating neuroinflamation, and regulating the associated molecular pathways

**DOI:** 10.1038/s41598-023-42485-4

**Published:** 2023-09-21

**Authors:** Muhammad Imran Khan, Eun Sun Jeong, Muhammad Zubair Khan, Jin Hyuk Shin, Jong Deog Kim

**Affiliations:** 1 Department of Biotechnology, Faculty of Biomedical and Life Sciences, Kohsar University, Murree, Pakistan; 2 Department of Laboratory Medicine, Yeosu Chonnam Hospital, Yeosu, Korea; 3 Department of Biotechnology, Chonnam Notational University, San96-1, Dun-Duk Dong, Yeosu, 59626 Chonnam Korea; 4 Research Center on Anti-Obesity and Health Care, Chonnam National University, San96-1, Dun-Duk Dong, Yeosu, 59626 Chonnam Korea

**Keywords:** Biochemistry, Biotechnology, Cell biology, Drug discovery, Neuroscience, Stem cells, Nanoscience and technology

## Abstract

Amyloid beta (Aβ) aggregation and tau hyper phosphorylation (p-tau) are key molecular factors in Alzheimer’s disease (AD). The abnormal formation and accumulation of Aβ and p-tau lead to the formation of amyloid plaques and neurofibrillary tangles (NFTs) which ultimately leads to neuroinflammation and neurodegeneration. β- and γ-secretases produce Aβ peptides via the amyloidogenic pathway, and several kinases are involved in tau phosphorylation. Exosomes, a recently developed method of intercellular communication, derived from neuronal stem cells (NSC-exos), are intriguing therapeutic options for AD. Exosomes have ability to cross the BBB hence highly recommended for brain related diseases and disorders. In the current study, we examined how NSC-exos could protect human neuroblastoma cells SH-SY5Y (ATCC CRL-2266). NSC-exos were derived from Human neural stem cells (ATCC-BYS012) by ultracentrifugation and the therapeutic effects of the NSC-exos were then investigated in vitro. NSC-exos controlled the associated molecular processes to drastically lower Aβ and p-tau. A dose dependent reduction in β- and γ-secretase, acetylcholinesterase, GSK3β, CDK5, and activated α-secretase activities was also seen. We further showed that BACE1, PSEN1, CDK5, and GSK-3β mRNA expression was suppressed and downregulated, while ADAM10 mRNA was increased. NSC- Exos downregulate NF-B/ERK/JNK-related signaling pathways in activated glial cells HMC3 (ATCC-CRL-3304) and reduce inflammatory mediators such iNOS, IL-1β, TNF-α, and IL-6, which are associated with neuronal inflammation. The NSC-exos therapy ameliorated the neurodegeneration of human neuroblastoma cells SH-SY5Y by enhancing viability. Overall, these findings support that exosomes produced from stem cells can be a neuro-protective therapy to alleviate AD pathology.

## Introduction

Alzheimer’s disease (AD) is characterized by a progressive impairment of memory and cognitive abilities^1^. The parenchymal deposition of amyloid-β (Aβ) plaques, the development of tau neurofibrillary tangles (NFTs), and neuroinflammation are histological indicators of AD^2,3^. AD patients eventually experience synapse loss and neuronal death, and the buildup of these lesions in the brain results in cognitive decline^4^. AD can cause patients to struggle with swallowing, walking, and communication as the disease progresses. Evidence suggests that tau protein and amyloid are leading causes of AD. Current research aims to develop medications that target tau and Aβ for the treatment of AD. The leading concepts in the development of senile plaques and NFTs involve the accumulation of Aβ proteins and hyperphosphorylated tau (p-tau), which inhibit microglial cell proliferation and cause neurodegeneration. One mechanism for the formation of Aβ proteins is the amyloidogenic breakdown of APP by β-secretase and γ-secretases, resulting in the formation of Aβ40 and Aβ 42^5^^.^ This process is thought to contribute to the pathogenesis of AD, which results in the increasing accumulation of A proteins^6,7^. The major elements of plaques in the brains of AD patients are the toxic fragments, Aβ42 and Aβ43^8^.

Increased β- and γ-secretase activity causes the creation and deposition of neurotoxic fragments Aβ42 and Aβ43.

These fragments lead to plaque formation and gradual neurodegeneration, ultimately resulting in AD. A disintegrin and metalloproteinase (ADAM), called α-secretase, breaks down the neuroprotein APP in the non-amyloidogenic pathway. Reducing Amyloid beta production is the most efficient method of treating AD, and this is achieved by activating α-secretase and inhibiting β- and γ-secretases.

The central nervous system’s neurons have high levels of tau, which stabilizes the microtubules that serve as motor protein tracks. Tau further modulates microtubule axonal transport^9–12^. Tau is increased to aid in the formation of new cell processes during neuronal development^13^. Paired helical filaments (PHFs), which collect to form NFTs, are formed when p-tau breaks off from the microtubules^14,15^. Tau is phosphorylated by a number of serine/threonine protein kinases, including glycogen synthase kinase-3 (GSK-3β) and cyclin-dependent kinase 5 (CDK5). Through priming kinases, like non-proline-directed kinases, GSK-3β increases the rate of tau phosphorylation by several fold^16–18^.

Exosomes derived from stem cells have attracted a lot of attention since they have various advantages over their parent cells, such as therapeutic potency, smaller size, simpler nature, and higher convenience in terms of production, handling, and storage. Additionally, exosomes have negligible immunogenicity and low tumorigenicity, and their membrane encapsulation shields the molecules they carry from deterioration^19–21^. Exosomes are saucer-shaped vesicles between 30 and 200 nm in size and are produced by endocytic processes. While exosomes were first classified as cellular debris carriers, they are now recognized as essential mediators of cell-to-cell transmission that act through the transfer of bioactive cargo, including proteins, RNA, and DNA^22–24^.

MicroRNA (miRNA), one example of exosomal cargo molecules, is crucial in mediating the therapeutic effects of these vesicles^25^. Nearly all body fluids, including blood, saliva, and urine, contain exosomes. Exosomes are released by cells under both pathological and normal physiological circumstances. Exosomes have become potential diagnostic and therapeutic agents due to these natural qualities^26^ and have been investigated in both experimental studies and clinical trials as biomarkers and therapeutic agents in a variety of illnesses^27^. Exosomes produced from mesenchymal stem cells (MSCs) have been proven in numerous studies to be able to lessen cognitive issues linked to a variety of neurological illnesses, including stroke, Parkinson's disease, and traumatic brain injury^28,29^^.^ Exosome release is thought to be a cellular adaptation mechanism, and its composition, biogenesis, and secretion will depend on the microenvironment with which cells interact^30^. It has been hypothesized that these vesicles act as paracrine activity effectors of MSCs by encapsulating and transferring many functional factors, including regulatory RNA, proteins, and lipids. When multivesicular endosomes fuse with the plasma membrane, a variety of cells naturally secrete exosomes, which further promote local or systemic cell-to-cell communication^31–33^. When under stress, such as during inflammation, exosomes may easily pass the blood–brain barrier (BBB)^34–36^^.^

In numerous mouse models of disease, including AD, administration of MSC-derived exosomes (MSC-exos) has been shown to increase neurogenesis in the subventricular zone (SVZ) and dentate gyrus (DG) of the hippocampus and enhance neuroprotection against inflammation and oxidative stress^37,38^. Therefore, research into the extensive therapeutic effects of MSC-exos on AD is worthwhile. Research objectives of the current study were to investigate the therapeutic effects of exosomes derived from neuronal stem cells (NSC-exos) on human neuroblastoma cells SH-SY5Y (ATCC CRL-2266) in Alzheimer's disease (AD) pathology. And to determine the underlying molecular mechanisms by which NSC-exos protect against AD pathology, including the regulation of Aβ and p-tau, reduction of inflammatory mediators, and modulation of associated molecular processes. In this study, we showed that Human neural stem cells (ATCC-BYS012) derived exosomes could function as a cell-free treatment for AD.

## Materials and methods

### Preparation of exosomes

Human neural stem cells (ATCC-BYS012) were cultivated in a full medium containing 1% exosome-free FBS (System Bioscience) for 48 h. At 90% cell confluency, condition medium was removed from cell cultures and filtered using a 0.22 µm Millipore filter (Bedford, MA, USA) to remove dead cells and big growth debris. The residual cell debris was then removed by centrifuging the medium at 1500 g for 30 min. Before purification, the collected supernatant was held on ice to prevent loss. ExoQuick-TC (System Biosciences) was used for exosome purification, which was followed by ultracentrifugation. Amicon® Ultra-15 Centrifugal briefly the supernatant was transfer to new tube and centrifuged it at 10,000 × g for 30 min at 4 °C followed by ultracentrifuge twice at 100,000 × g for 90 min at 4 °C using a swinging bucket rotor the supernatant and collect exosome pellets by resuspending in 1 mL of PBS. Exosome were processed for further experiments. Filter Units of 100 kDa (Millipore, Bedford, MA, USA) were used to concentrate the filtered supernatant. Nanoparticle tracking analysis (NTA) and atomic force microscopy (AFM) were used to measure the shape and size of exosomes^39^. Briefly 10 μL sample of exosomes were 1000 thousand times diluted and processed for NTA and AFM. Western blotting (WB) was used to detect the exosome-associated marker CD63 (CBL553, Millipore, Bedford, MA, USA). An ExoView Tetraspanin chip assay was also used to characterize the isolated exosomes.

### Enzyme activity analysis by fluorometric methods

The effects of NSC-exos on the production of Aβ and p-tau and the activities of secretases, and phosphorylating kinases, such as GSK-3β and CDK5, were assessed using a specially designed ELISA kit. According to the manufacturer’s recommendations, enzyme activity was tested using the SensoLyte®520 kit appropriate to each enzyme. Six-well plates with cells were grown (0.3 × 10^6^ cells/well) and incubated at 37 °C with 5% CO_2_. Cells were given NSC-exos treatment after attachment after 2 h and were kept under exosomes treatment for 24 h. Cells that were not treated served as the control. Each enzyme-specific assay required the preparation and usage of cell lysate. Using an ELISA microplate reader, absorbance was measured at 450 nm. The manufacturer’s recommendations were followed while analyzing and comparing the data. Three duplicates of each experiment were conducted.

SH-SY5Y Human Neuroblastoma Cell Line (ATCC CRL-2266) were obtained from ATTC and were cultured in 6-well plates (0.3 × 10^6^ cells/well) and exposed to various concentrations (5–15 μL) of NSC-exos treatment for 24 h to measure the activity of acetylcholinesterase. For the experiments, cells were collected, and lysate was added to 96-well microplates. Using a microplate reader, absorbance was measured at 450 nm. The manufacturer’s recommendations were followed while analyzing and comparing the data. Acetylcholinesterase Activity Inhibitory Assay The manufacturer's instructions were followed while using an acetylcholinesterase activity assay kit (Sigma Aldrich) to measure the inhibition of acetylcholinesterase activity.

### Determination of the levels of Aβ42 and phosphorylated tau (p-tau) using ELISA

The effects of NSC-exos on the expression levels of p-tau and Amyloid beta, which are linked to AD, were assessed using specific ELISA kits in accordance with the manufacturer’s recommendations. NSC-exos were applied to SH-SY5Y cells that were grown in 6-well plates. For the experiments, cells were collected, and lysate was added to 96-well microplates. Using a microplate reader, absorbance was measured at 450 nm. In accordance with the manufacturer’s recommendations, data were examined and contrasted.

### RT-PCR analysis

SH-SY5Y cells were grown in 6-well plates with DMEM medium supplemented with 10% FBS and incubated at 37 °C in a CO_2_ incubator. Following NSC -exos treatment, these cells were cultured for 24 h. Using an RNA isolation kit (Sigma Aldrich, Cat. No. 83913) and DNase treatment, total RNA was recovered from the cells. A NanoDrop 2000/2000c spectrophotometer (Thermo Fisher Scientific, Wilmington, DE, USA) was used to quantify the extracted RNA. With the use of the Revert Aid Premium First Strand cDNA Synthesis Kit (Thermo Fisher Scientific No. K1621), the RNA (500 ng) was then reverse-transcribed into cDNA. With the use of a Thermo Scientific qPCR kit, cDNA was processed for real-time qPCR with gene-specific primers after being measured with the NanoDrop spectrophotometer. The list of primers used is mentioned in Table 1.

**Table 1:** Primer sequences used in RT-PCR.

No	Gene	Primer sequence	Accession number	Size [bp]
1	BACE	GAGTCCCTCACGCTGCAAAG TGGAGAGTGGGCAGGAGAAATC	AF190726	199
2	PSEN1	GTGTTCTACTTCGCCACGGA GCGATGGATGTTGGAAACCG	NM_001362271	297
3	Acetylcholinesterase	GCATACACCTTCCCTGGCTT CTTGGGCTCTGGTGGCATAA	X56518	229
4	GSK-3β	AGCTCTGATTGGCCACTGTC TGGGAAGGAGGGAGGAGATG	NM_019827	936
5	CDK5	TGAGGGTGTGCCAAGTTCAG GCATTAGCCAGCTCTCAGCA	NR_156449	374
6	ADAM10	CAAAAACACCAGCGTGCCAA ATGCTTCTCTGGATGTGCCC	NM_007399	474
7	NF-KB	ACACATCCGGACCTCGCA TCTGAAGCTCTCTCCTCCGC	NM_001319226.2	200
8	MAPK/ERK	ACTCACTTCACCAGGATGCG GCTGTCCTGTTGACCATCCA	NM_005921.2	111
9	Actin	CGCCAGCCTCTGAAACTAGA ATAGATGGGCACGTTGTGGG	NM_001272041	528

BACE, β-secretase; PSEN1, presenilin 1 (γ-secretase); GSK-3β, glycogen synthase kinase-3; CDK5, cyclin-dependent kinase 5; ADAM10, A disintegrin and metalloproteinase domain-containing protein 10 (α-secretase); bp, base pairs.

### Western blotting

NSC-exos were administered to SH-SY5Y cells after they had been cultured and reached confluency. Cells were treated, then lysed on ice with radio immunoprecipitation assay buffer and rinsed with PBS buffer (Sigma-Aldrich). The supernatant, which contained the proteins, was collected after centrifuging and sonicating the lysed cells. Using the BCA protein assay (Pierce), protein content in the supernatant was determined. The electrophoresis of 10% sodium dodecyl sulfate polyacrylamide gel was used to separate 50 µg of each total protein. Proteins were transferred to nitrocellulose membranes (Bio-Rad). The membrane was blocked in 5% (w/v) non-fat dried milk in Tris-buffered saline (TBS) for one hour at room temperature after being washed with double-distilled water. After that, the membranes were incubated with p-tau-specific primary antibodies diluted in TBS with 5% non-fat dried milk for two hours at 4 °C. Protein bands were visible through chemiluminescence after each membrane had been rinsed with double-distilled water and treated for two hours with a secondary antibody coupled to horseradish peroxidase (1:1000, Pierce Biotechnology). The protein bands were quantified and normalized using Fluor-S MultiImager TM software (Bio-Rad in Hercules, California, USA). The same experiment was run twice.

### Cell viability

The viability of the SH-SY5Y cells with and without exosome treatment was measured using LIVE/DEAD™ Viability/Cytotoxicity Kit (cat# L3224, Invitrogen). Briefly, exosome treated cells washed using DPBS, followed by incubation with dead/live solution for 20 min at 37 °C. After incubation, cells were washed and visualized under microscope.

### Measurement of proinflamatory cytokines production

iNOS, IL-1β, TNF-α, and IL-6 were measured in activated glial cells HMC3 (ATCC-CRL-3304) using kits from R&D Systems (Minneapolis, MN, USA). In a nutshell, the cell culture's supernatant was gathered and centrifuged. For ELISA, samples were placed in each well. Each sample’s concentration was determined using the standards included in the kits. Effects on the suppression of NF-Kb and ERK was assessed by RT-PCR.

### Statistical analysis

GraphPad Prism 8 (version 8.2.0 GraphPad Software Inc., Hercules, CA, USA) was used for the statistical analysis. The Kolmogorov–Smirnov (K-S) and Levene’s tests were used to assess the normality and homogeneity of the data, respectively. With a *p*-value of 0.162, the data are somewhat regularly distributed. Tukey’s post hoc analysis was used to analyze one-way ANOVA results in order to compare the statistical significance of the results between the various groups. The level of significance was held at 0.05. Means were computed after experiments were run in triplicate.

## Results

### Isolation of exosomes from stem cells

**Figure 1:**
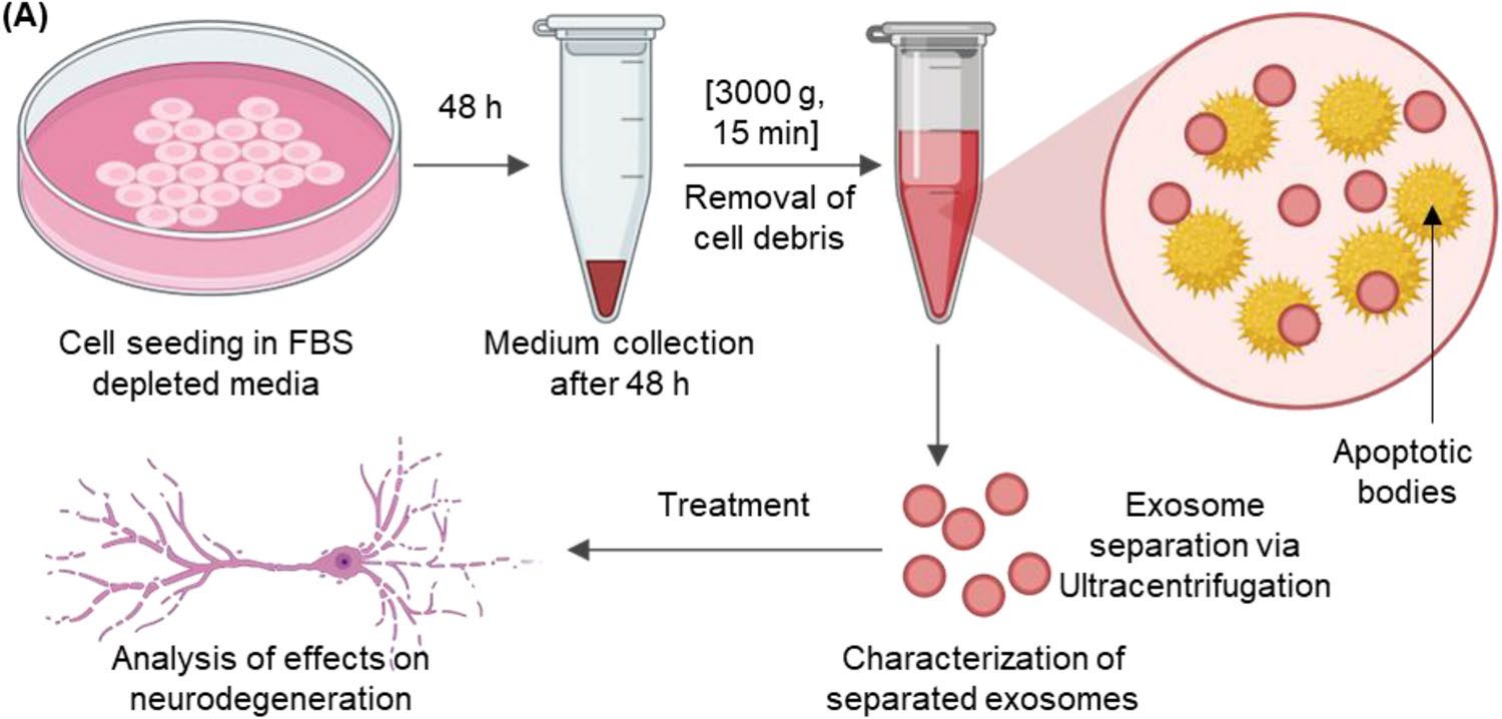
Flowchart illustrating the experimental process. Cells were seeded in cell media containing exosome-depleted FBS for 48 h. After 48 h, cell-conditioned media was collected and centrifuged at 3000 g for 15 min to remove cell debris. NSC-exos were separated using centrifugation and separated NSC-exos were characterized for further analysis. Finally, NSC-exos were administered to the neurodegenerative disease cell culture model to analyze their effects.

In a 6-well plate, stem cells were grown, and NSC-exos were produced by ultracentrifuging the cells to separate them. In Fig. 1, the flowchart illustrates this process. NSC -exos were extracted and prepared in sufficient quantities for the studies.

### Characterization of isolated exosomes

**Figure 2:**
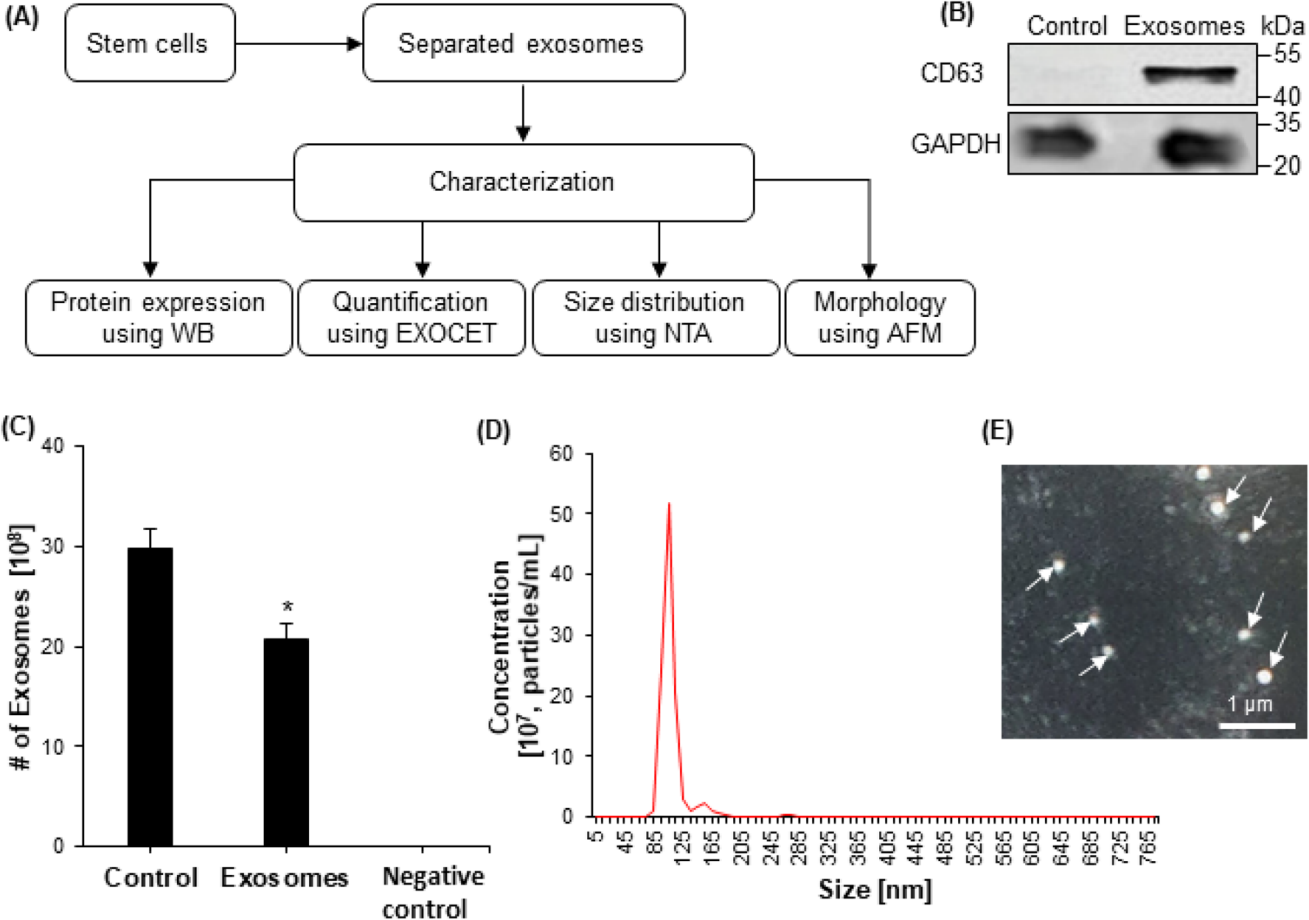
Characterization of exosomes. (**A**) A flowchart indicated NSC-exos were separated using centrifugation and characterized for protein expression using WB, size distribution using NTA, and quantification using EXOCET. (**B**) Protein expression was measured using WB for exosome marker CD63 in control, only cell-free media, and in separated NSC-exos. GAPDH was used as an internal control. (**C**) Exosomes were quantified using an EXOCET assay in control (unprocessed cell-conditioned media), NSC-exos, and negative control. (**D**) Concentration of separated exosomes was measured using NTA. The highest peak was observed at 105 nm. (**E**) Morphology of separated exosomes was visualized using AFM. The circular morphology was easily observed.

NTA, WB, AFM, and ELISA were used to characterize the NSC-exos (Fig. 2A). The isolated NSC-exos also expressed CD63 tetraspanin shown by WB (Fig. 2B). Exosomes were quantified using an EXOCET assay in control (unprocessed cell-conditioned media), NSC-exos, and negative control (Fig. 2C). The NTA results showed that exosomes with a size range of 30–200 nm were present (Fig. 2D).AFM further verified the exosome size range of 30–150 nm (Fig. 2E). ELISA uses the surface marker proteins to identify exosomes.

### Effects of exosomes on the activities of various enzymes

**Figure 3:**
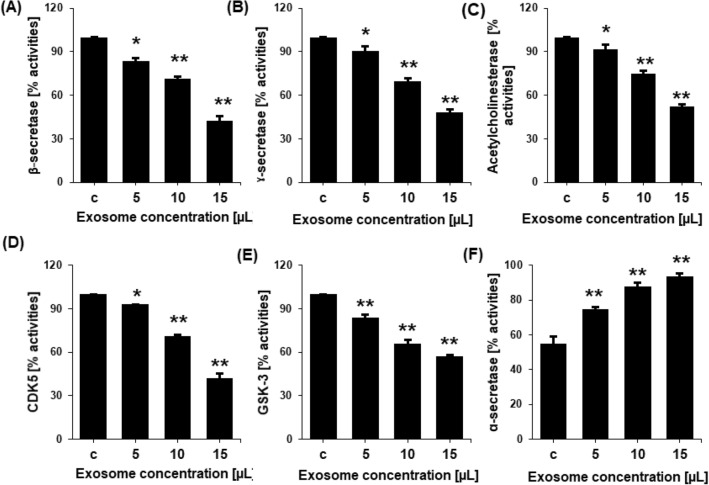
Inhibition of various enzymes involved in neurodegeneration leading to processing and production of p-tau and Aβ. Inhibition of the percentage of activities of (**A**) β-secretase (**B**) γ-secretase, (**C**) acetylcholinesterase, (**D**) CDK5, and (**E**) GSK-3 in untreated cells (control) or cells treated with 5, 10, or 15 µL NSC-exos. (**F**) The percentage activity of α-secretase increased in NSC-exos-treated conditions. Data is expressed as the mean ± standard error of the mean (SEM) from three independent experiments (n = 3). * = p < 0.01 and ** = p < 0.001.

The in vitro activity of the enzymes that produce A and p-tau, including acetylcholinesterase, β-secretase, γ-secretase, α-secretase, GSK-3, and CDK5, were shown to be regulated by exosomes. Different nontoxic concentrations of the recovered NSC-exos were applied to SH-SY5Y cells, and the cell lysate was then prepared for ELISA. The activity of the enzymes acetylcholinesterase, β-secretase, γ-secretase, GSK-3, and CDK5 were found to be dramatically reduced by exosomes in a dose-dependent manner at various significant levels (Fig. 3A–E). NCS-exos were also shown to improve the actions of α-secretase (Fig. 3F). When compared to the levels in the control, the ELISA showed that these proteins were present at dose-dependently higher levels.

### RT-PCR analysis

**Figure 4:**
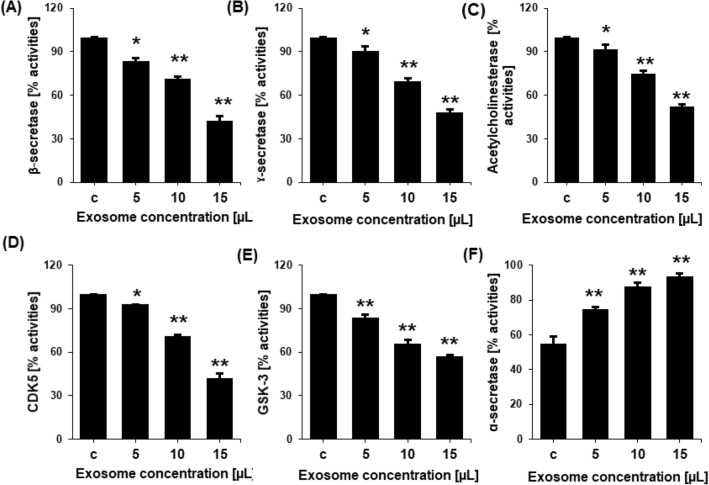
mRNA expression level of various secretases and kinases involved in neurodegeneration and processing of Aβ and p-tau leading to plague and NFTs in the precence of various concentrations of NSC-exos. qRT-PCR results revealed the suppression of (**A**) BACE1, (**B**) PESN, (**C**) acetylcholinesterase, (**D**) GSK-3β, and (**E**) CDK5 mRNA expression, and the enchancment of (**F**) ADAM10 mRNA expression. Data is expressed as the mean ± standard error of the mean (SEM) from three independent experiments (n = 3). * = p < 0.01 and ** = p < 0.001.

qRT -PCR was used to measure the expression of genes involved in the Aβ pathway using gene-specific primers (Table 1). The results revealed a significant effect of exosome treatment on the expression of α-secretase (ADAM10). The expression level of ADAM10 was upregulated in a dose-dependent manner. Exosome treatment significantly decreased the expression of the β-secretase (BACE1), γ-secretase (PS1), GSK-3β, and CDK5 at various levels (Fig. 4).

### Determination of Aβ and p-tau levels using ELISA

**Figure 5:**
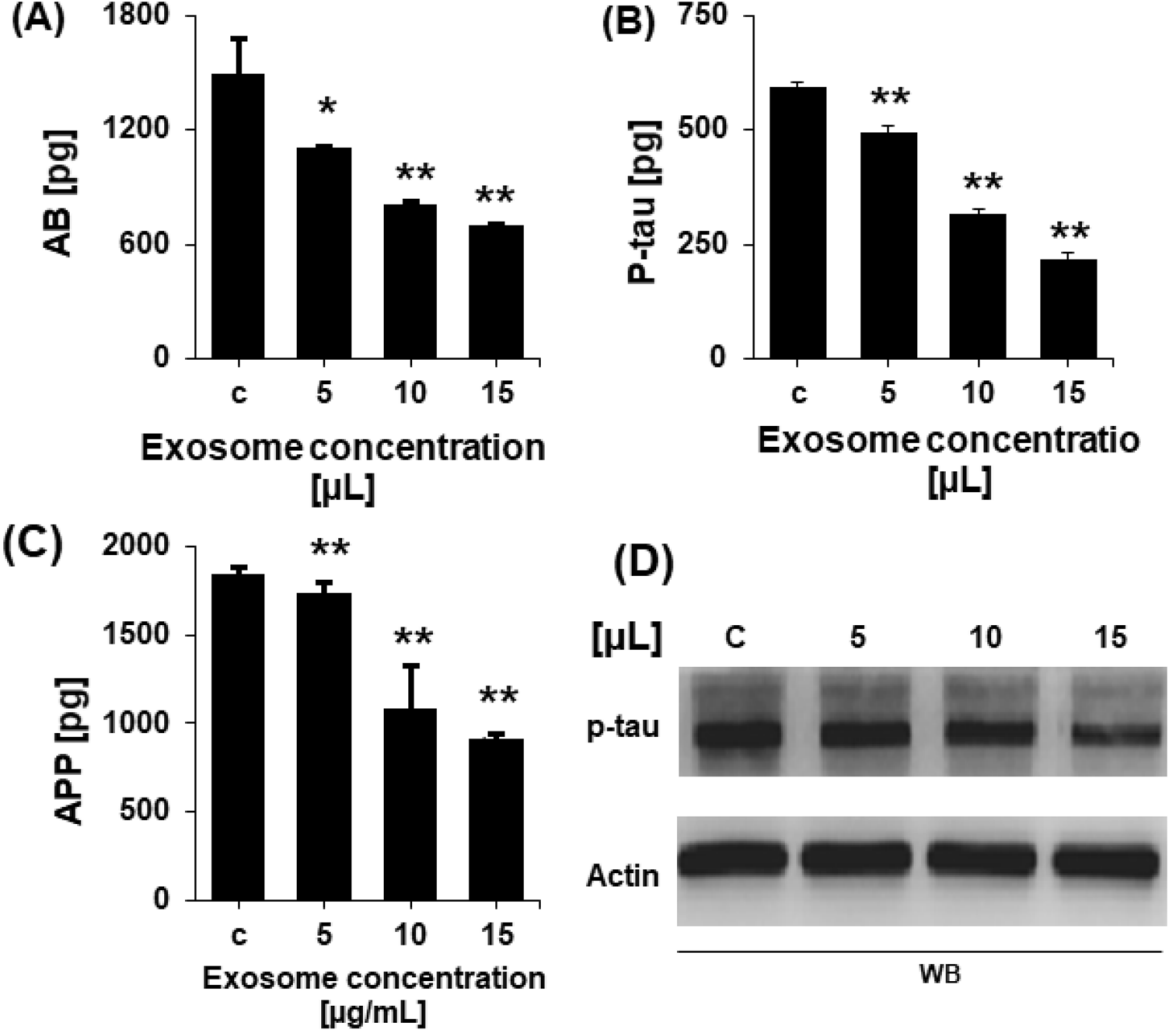
Concentration level of Aβ and p-tau in non-treated and NSC-exos-treated SH-SY5Y cells determined by ELISA and WB. Concentration level of (**A**) Aβ, (**B**) P-tau, and (**C**) APP decreased in NSC-exos-treated cells in a dose-dependent manner. No effect was observed in non-treated cells. (**D**) Concentration of p-tau was measured in control and NSC-exos-treated cells. β-actin was used as an internal control. Data shown here as the mean ± standard error of the mean (SEM) from three independent experiments (n = 3). * = p < 0.01 and ** = p < 0.001.

After determining the effects of exosomes on the genes involved in AD alleviation and AD pathogenesis, the Aβ and p-tau levels in cells after NSC-exos treatment were measured using specific ELISA kits. p-tau were measured by WB (Full length uncroped images of WB gels are provided in Supplementary Files). The levels of p-tau and Aβ were significantly and dose-dependently reduced in treated cells compared to those in the non-treated cells (Fig. 5). These findings demonstrate the effects of NSC-exos on the inhibition of p-tau and Aβ accumulation- and promotion-associated genes and the enhanced expression levels of the AD alleviating genes.

### Therapeutic effects of chromosomes on neurons degeneration

**Figure 6:**
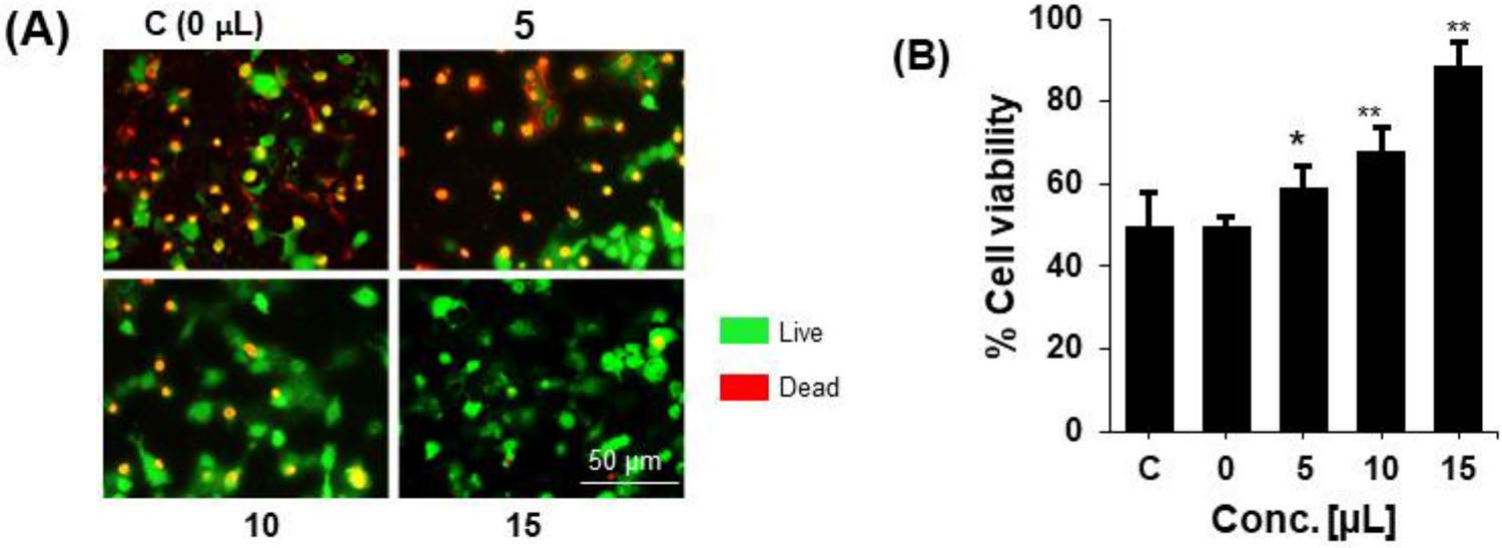
Effect of NSC–exos on cell viability of SH-SY5Y cells. (**A**) Representative stained images and (**B**) quantification of NSC–exos treatment of cells. Cells were treated with Aβ42 and then with NSC–exos in a dose-dependent manner. Cells showed increased viability with increased NSC–exos concentration, while more dead cells were observed in non-treated cells. Data is pressented as the mean ± standard error of the mean (SEM) from three independent experiments (n = 3). * = p < 0.01 and ** = p < 0.001.

The protective effects of NSC-exos were determined by measuring the viability of the SH-SY5Y cells cultured and treated with various concentrations of NSC-exos for 72 h. The dead or degenerated cells were determined by viability using a live and dead cell detecting kit. It was found that NSC-exos treatment increased the viability of cells dose-dependently (Fig. 6). These therapeutic effects might be due to the regulation of the enzymes and genes in the SH-SY5Y cells by NSC-exos treatment.

### Determination of Aβ and p-tau levels using ELISA

**Figure 7:**
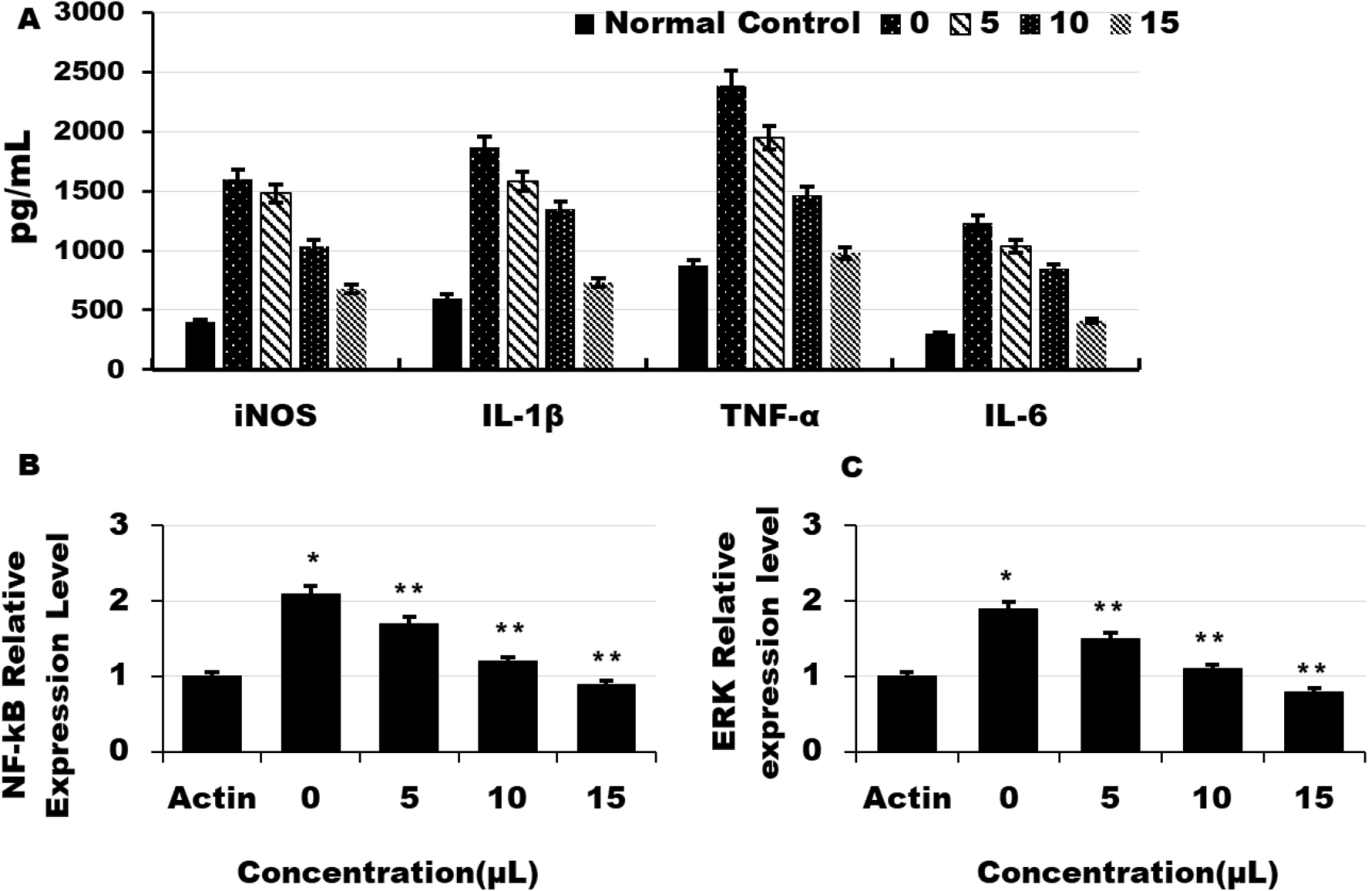
Effect of NSC–exos on proinfmatory cytokines and mRNA exoression of the genes of kinases and transcriptional factors leading to neuroinflamtion in glial cells. Actvated Glial cells were treated with NSC- -exos in and the proinflamatory cytokines were quantified and expression level of the AMPK/ERK and NF- kB were analysed. (**A**) Quantifification of iNOx, IL-1B,TNF-alpha and IL-6 in glial cells of control and treated groups**. **(**B**) mRNA expression level of NF-kB in verious groups cells (treated and controls) quantified by qRT-PCR. (**C**) mRNA expression level of NF-kB in verious groups cells (treated and controls) quantified by qRT-PCR. Data is pressented as the mean ± standard error of the mean (SEM) from thre independent experiments (n = 3). * = p < 0.01 and ** = p < 0.001.

In order to check the effects of NSC-exo on neuroinflamation, glial cells HMC3 were culterd and treated with LPS to actvate and then treated with verious concentrations of exosomes. Proinflamtory cytokines were then quantified in traeted and control cells groups. The results showed a good effects of the exosomes on the reduction of the verious inflamation causing cytokines e.g. iNOS, IL-1β, TNF-α, and IL-6 in dose dependent manner. The mRNA expression level of the MAPK/ERK and NF-kB were also downregulted after treatment with exosomes as compared to non-treated control in dose dpentdant manner (Fig. 7).

## Discussion

The most prevalent neurodegenerative illness, AD, is defined by the death of neurons and the impairment of memory, cognition, and daily living skills. Death frequently results from impairment and the loss of fine motor abilities^40–42^. The formation of NFTs, which are made up of p-tau protein, and the accumulation of Aβ plaques are the primary pathological indicators of AD^43^. Early on, the medial temporal lobe is the primary site of these degenerative alterations, which thereafter disseminate across the neocortex^44^. Due to its ongoing breakdown of the neurotransmitter acetylcholine, acetylcholinesterase has been determined to be a progressive cause of AD. Due to the formation of A, α7 nAChR, the most prevalent subtype of acetylcholine receptor in the brain, is drastically decreased in AD patients. Neprilysin, acetylcholinesterase, Aβ generation, and α, β, and γ-secretases as well as other key molecular targets, are therefore essential for AD therapeutic drug screening. SH-SY5Y, as in vitro modules, are a promising choice for examining the therapeutic effects of medicines on modulation of APP processing and A-induced AD pathogenesis, but this model restricts the study of other hypotheses and molecular targets accountable for AD pathogenesis.

Different kinases produce hyperphosphorylation of tau at diverse sites. In healthy individuals, tau kinase and tau phosphatase activity are in equilibrium. This event is crucial for decreasing tau’s affinity for microtubules and boosting tau’s resistance to calcium-activated neutral proteases and ubiquitin-proteosome-mediated tau degradation^47^^.^ Hyperphosphorylation, which results from an imbalance in tau phosphorylation, causes tau to fibrillize and aggregate, creating NFTs^45–50^. GSK3β, CDK5, calmodulin-dependent protein kinase II (CaMK II), and microtubule affinity regulating kinase (MARK) are a few of the most important tau kinases^51–55^. It is well known that AD and other associated illnesses alter tau kinase and tau phosphatase expression and activity^56,57^^.^

NSCs have neuroprotective paracrine actions that can stimulate neuronal development, proliferation, and survival in endogenous neurogenic niches in cellular models of AD^58,59^. NSC transplantation has been reported to minimize a deposition, induce neurogenesis, and ameliorate memory and spatial learning deficits in mouse AD models^60^. Since mass neuronal and synaptic loss characterizes AD as a neurodegenerative condition, regeneration of neuronal circuits by exogenous NSCs is a promising therapeutic approach^61^. However, there is still controversy around the hazards of tumor development, immunological rejection, and infusion toxicity in NSC transplantation. NSC-exos therapy has potential for treating Alzheimer's disease, but the possibility of off-target effects, adverse reactions, and long-term safety concerns must be considered. Immune reactions and inflammation may occur in response to the exosomes, and unintended targeting of healthy cells or tissues could lead to adverse effects. Additionally, the long-term safety of exosomes therapy is uncertain, so further research is needed to assess its potential risks and benefits. As a cell-free therapy, NSC-exos appear to be as successful as NSCs, and according to mounting evidence, NSC -exos are also less immunogenic than their parent cells, easier to generate and store, and do not possess tumorigenic properties^62,63^.

In the current study, we separated exosomes from stem cells and examined their impact on Aβ, the main contributor to neurodegeneration in AD. Our findings showed that NSC-exos dramatically reduced Aβ by stimulating APP processing by the non-amyloidogenic route vis raising ADAM10 (α-secretase) activities and by inhibiting BACE1 (β-secretase) and PESN1 (γ-secretase). NSC-exos also greatly reduced the amyloidogenic cleavage of APP. By reducing the expression and activity of BACE1 and PESN1, the synthesis of amyloidogenic Aβ have been prevented We discovered that, following NSC-exos treatment, BACE1 and PESN1 expression levels were all decreased in a dose-dependent manner whereas ADAM10 expression levels were increased. The increased non-amyloidogenic APP processing by ADAM10 and the decreased amyloidogenic processing by BACE1 and PSEN1, which is supported by the decline in Aβ level, may be the causes of the Aβ reduction. Overall, NSC-exos reduced the generation of Aβ while also activating a non-amyloidogenic route and suppressing the amyloidogenic pathway. For the prevention or reduction of neurodegeneration and AD, Aβ production must be inhibited or reduced. NCS-exo alleviate neuroinflamtion which is the major cause of neurodegeneration and Alzheimer pathogenesis by suppressing the expression of the proinflamatory cytokines and molecular pathways. The study has some limitations as it relies on in vitro models, further in vivo validation studies are needed to confirm the potential therapeutic effects of NSC-exos on AD pathology.

## Conclusions

In the present study, we revealed that NSC-exos is a promising therapeutic for the treatment and prevention of AD. NSC-exos therapy reduced p-tau levels and Aβ formation via the suppression of kinase expression and activity, and AD pathology-promoting genes and proteins. Also the NC- Exo reduced neuroinflamation. Further studies and clinical trials are required to incorporate the NSC-exos in pharmaceutical formulations.

### Supplementary Information

Supplementary Information.

## Data Availability

Data are under use for research purposes; however, data will be provided on proper request to the corresponding author.

## Sample availability

Samples are available from the authors on proper request.
