# Supplementary figures and images for: Stem cells-derived exosomes alleviate neurodegeneration and Alzheimer’s pathogenesis by ameliorating neuroinflamation, and regulating the associated molecular pathways
Source: Sci Rep. 2023 Sep 21;13:15731. doi: 10.1038/s41598-023-42485-4 (PMC10514272; doi:10.1038/s41598-023-42485-4)

## Full/Uncropped/ gel pictures

Actin

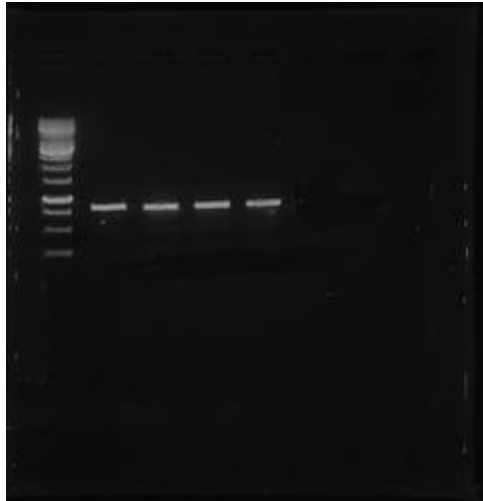

ADAM 10

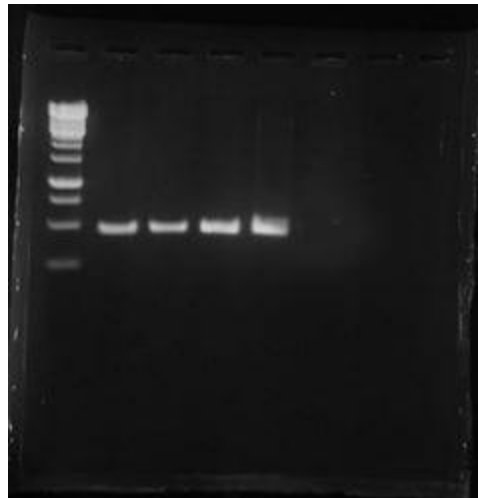

BACE1

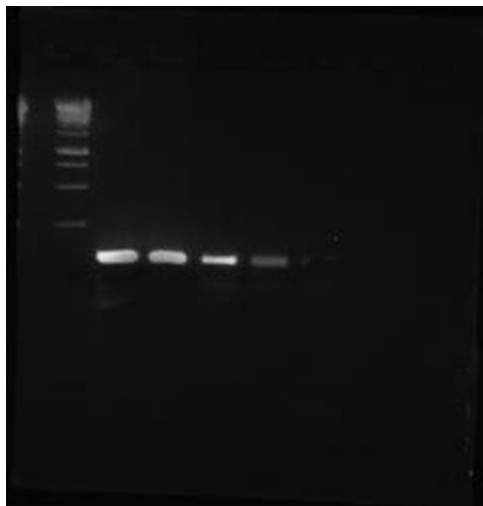

PESN1

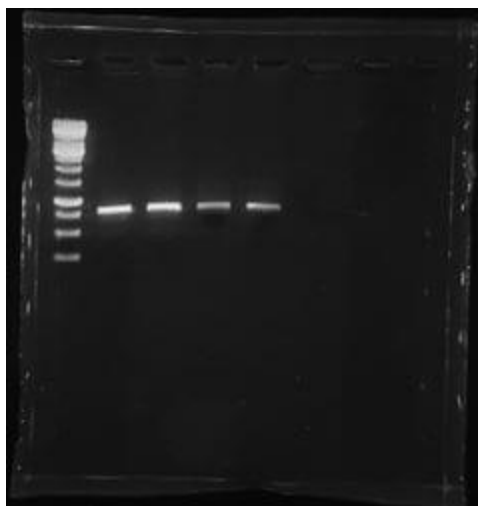

Acetyl cholinesterase

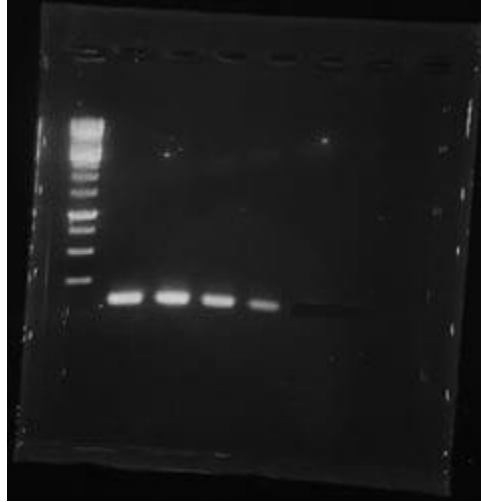

p-tau

Actin

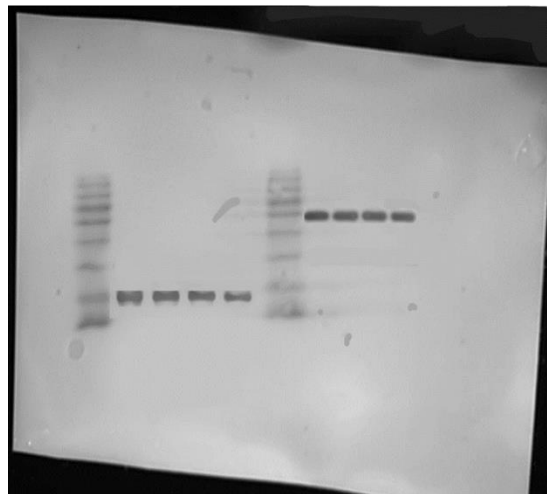

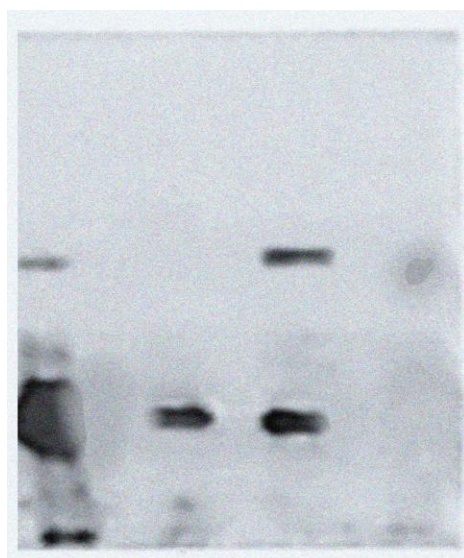

CD63

GAPDH

Supplement: Supplementary file 1 — Supplementary Information. [file 41598_2023_42485_MOESM1_ESM.pdf]
